# Segregation of Ca^2+^ signaling in olfactory signal transduction

**DOI:** 10.1085/jgp.202213165

**Published:** 2023-02-14

**Authors:** Hiroko Takeuchi, Takashi Kurahashi

**Affiliations:** 1 Department of Biophysical Dynamics, Graduate School of Frontier Biosciences, Osaka University, Osaka, Japan

## Abstract

Olfactory signal transduction is conducted through a cAMP-mediated second messenger cascade. The cytoplasmic Ca^2+^ concentration increases through the opening of CNG channels, a phenomenon that underlies two major functions, namely, signal boosting and olfactory adaptation. Signal boosting is achieved by an additional opening of the Ca^2+^-activated Cl^−^ channel whereas adaptation is regulated by Ca^2+^ feedback to the CNG channel. Thus, the influx of Ca^2+^ and the resultant increase in cytoplasmic Ca^2+^ levels play seemingly opposing effects: increasing the current while reducing the current through adaptation. The two functions could be interpreted as compensating for each other. However, in real cells, both functions should be segregated. Ca^2+^ dynamics in olfactory cilia need to be directly measured, but technical difficulties accompanying the thin structure of olfactory cilia have prevented systematic analyses. In this study, using a combination of electrophysiology, local photolysis of caged cAMP, and Ca^2+^ imaging, we found that free Ca^2+^ in the local ciliary cytoplasm decreased along with a reduction in the current containing Ca^2+^-activated Cl^−^ components returning to the basal level, whereas Ca^2+^-dependent adaptation persisted for a longer period. The activity of Cl^−^ channels is highly likely to be regulated by the free Ca^2+^ that is present only immediately after the influx through the CNG channel, and an exclusive interaction between Ca^2+^ and Ca^2+^-binding proteins that mediate the adaptation may modulate the adaptation lifetime.

## Teaser

In olfactory transduction, Ca^2+^ signaling is used for two opposing functions, namely signal boosting and signal reduction through adaptation, which could be interpreted to compensate for each other. Here, however, we show that these functions are clearly segregated by molecular dynamics.

## Introduction

Olfaction begins in the sensory cilia of olfactory receptor cells (ORCs). Olfactory cilia exhibit a fine cylindrical structure with a 100–200-nm diameter and measuring several tens of micrometers in length (Usukura and Yamada, 1978; Lidow and Menco, 1984; Morrison and Costanzo, 1992). Within the cilia, signal transduction occurs through a cAMP-mediated second messenger cascade (Nakamura and Gold, 1987; Kurahashi, 1990, see reviews of Pifferi et al., 2010; Boccaccio et al., 2021). In addition, the cytoplasmic Ca^2+^ concentration ([Ca^2+^]_i_) in the cilia increases (Leinders-Zufall et al., 1997) through the opening of CNG channels, which underlies two major functions, namely signal boosting (Kleene, 1993; Kleene, 1999; Kurahashi and Yau, 1993; Lowe and Gold, 1993) and olfactory adaptation (Kurahashi and Shibuya, 1990). Signal boosting is achieved by an additional opening of the Ca^2+^-activated Cl^−^ channel (Takeuchi and Kurahashi, 2018), whereas olfactory adaptation, especially short-term adaptation, is regulated by Ca^2+^ feedback to the CNG channel (Kurahashi and Shibuya, 1990; Chen and Yau, 1994; Kurahashi and Menini, 1997). Thus, the influx of Ca^2+^ and the resultant increase in cytoplasmic Ca^2+^ levels play seemingly opposing effects: increasing the current while reducing the current through adaptation. These functions could be interpreted seemingly to compensate for each other, but they should be segregated in real cells. It has been shown in previous research that second messengers operate within a limited area around the vicinity of the signal origin (Takeuchi and Kurahashi, 2018). Therefore, the opposing functions by Ca^2+^ molecules are not isolated spatially but occur in the same restricted area.

Specifically, the reason why the activity of Ca^2+^-activated Cl^−^ channels is reduced while Ca^2+^-dependent adaptation is maintained (see Kurahashi and Shibuya, 1990; Kurahashi and Menini, 1997) is puzzling. Possibly, the Cl^−^ channels may show desensitization when situated in the native cilia. Another possibility is the long-term effects of Ca^2+^ on adaptation systems. It is essential to observe the change in Ca^2+^ activities in the local area of the cilium while measuring the activities of transduction channels to solve these problems. However, technical difficulties inherent in the thin structure of olfactory cilia have prevented systematic analyses. In this study, we visually monitored Ca^2+^ dynamics in highly localized areas of single cilia using a Ca^2+^-sensitive dye that was introduced into the ciliary cytoplasm from the whole-cell (WC) recording pipette. By simultaneously monitoring membrane currents, we could set the stimulus strength within the dynamic range of the signal transduction machinery and directly compare the profiles of the currents with the dynamics of the [Ca^2+^]_i_ changes. [Ca^2+^]_i_ locally (<1 μm) increased upon the opening of the CNG cation channels, which was mediated by laser photolysis of cytoplasmic caged cAMP. We also observed that the Ca^2+^ signal returned to the basal level after the termination of local UV stimuli with a time course similar to that for the cAMP-induced current that contained a Cl^−^ component. By contrast, Ca^2+^-dependent adaptation persisted within the same restricted area for a longer period. It is generally known that the WC recording configuration needs to use exogenous Ca^2+^ buffers to maintain a low cytoplasmic Ca^2+^ level. However, extrinsic EGTA may cause side effects that are distinct from the endogenous Ca^2+^ buffers, especially when the focus is on the kinetics of Ca^2+^. In this study, we also succeeded in recording cell responses without adding exogenous Ca^2+^ buffers, presumably before the intrinsic Ca^2+^ buffers were washed out, and we observed essentially the same results. It was indicated in the results that the seemingly two opposing functions of Ca^2+^ are clearly segregated by molecular dynamics, even in submicron spaces in native olfactory cilia. The activity of Cl^−^ channels is highly likely regulated by free Ca^2+^ and is only available immediately after the influx through the CNG channel. In addition, an exclusive interaction between Ca^2+^ and Ca^2+^-binding proteins that mediate the adaptation may modulate the adaptation lifetime.

## Materials and methods

### Ethical approval

The experiments were conducted under the Osaka University Regulations on Animal Experiments established by the Animal Experiment Committee at Osaka University in Japan. Approval number: FBS-19-002. Date of approval: May 20, 2019.

### Cell dissociation

The method was essentially the same as in previous studies (Kurahashi, 1989; Takeuchi and Kurahashi, 2005, 2018). Briefly, the cells were dissociated from the epithelium of the newt (*Cynops pyrrhogaster*) because of considerable ORC sizes. The animals were chilled on ice and double pithed. After decapitation, the olfactory epithelia were removed and incubated at 37°C for 5 min in 1% collagenase media containing (in mM) 110 NaCl, 3.7 KCl, 10 HEPES, 15 glucose, 1 pyruvate, and 0.001% phenol red, with pH adjusted to 7.4 using NaOH. Then, the ORCs were mechanically isolated by trituration using a 5-ml measuring pipette. The cells were adhered onto the surface of concanavalin A-coated glass coverslips and placed at the bottom of Petri dishes. The cells were maintained at 4°C before use in normal Ringer’s solution containing (in mM) 110 NaCl, 3.7 KCl, 3 CaCl_2_, 1 MgCl_2_, 10 HEPES, 15 glucose, 1 pyruvate, and 0.001% phenol red, with pH adjusted to 7.4 using NaOH.

### Electrophysiology

Ciliary membrane currents were recorded from single ORCs with a WC recording configuration (Hamill et al., 1981) under the voltage-clamp mode (V_h_ = −54 mV) as previously described (Takeuchi and Kurahashi, 2002; 2019). The culture dish was mounted on the stage of laser scanning microscopy (LSM, Axiovert 510 system; Carl Zeiss Microimaging GmbH). Patch pipettes (resistance, 10–15 MΩ) were made from borosilicate tubing with filaments (outer diameter, 1.2 mm; World Precision Instruments) using a two-stage vertical patch electrode puller (PP-830; Narishige). The recording pipette was filled with Cs^+^ solution containing (in mM) 119 CsCl, 1 CaCl_2_, 5 EGTA, 10 HEPES, and 0.001% phenol red (pH 7.4 adjusted using CsOH), as well as 1 mM caged cAMP (catalog number 116810; Calbiochem; Merck Millipore) and 50 µM Fluo-4 (F14200; Invitrogen; Thermo Fisher Scientific). Current signals were I-V converted using a 200B amplifier (Molecular Devices LLC), and data were sampled using pCLAMP ver.10 (Molecular Devices LLC) at 10 kHz, after being low-pass filtered at 2 kHz. For curve drawings of the membrane current, some data were low-pass FFT-filtered at 0.02 kHz. Care was taken to avoid saturation of response, particularly when evaluating adaptation. All experiments were performed at room temperature (23–25°C).

### Photolysis of the caged compound

For the stocks, caged cAMP was dissolved in DMSO (Takeuchi and Kurahashi, 2002; Takeuchi and Kurahashi, 2008; Takeuchi et al., 2013; Takeuchi and Kurahashi, 2018) and stored at −20°C under complete darkness (for up to 180 d). After the WC recording configuration was established, the caged compounds were introduced to the cell interior through free diffusion. The UV laser beam (80 mW: Argon laser λ = 351, 364 nm; Coherent) was used to photolyze the caged cAMP. For local spot UV photolysis in a single cilium, the region of interest (ROI) function of an LSM was used for local and spatially restricted stimulation, as previously described (Takeuchi and Kurahashi, 2008).

### ROIs for image scans and UV stimulation

**Figure 1.**
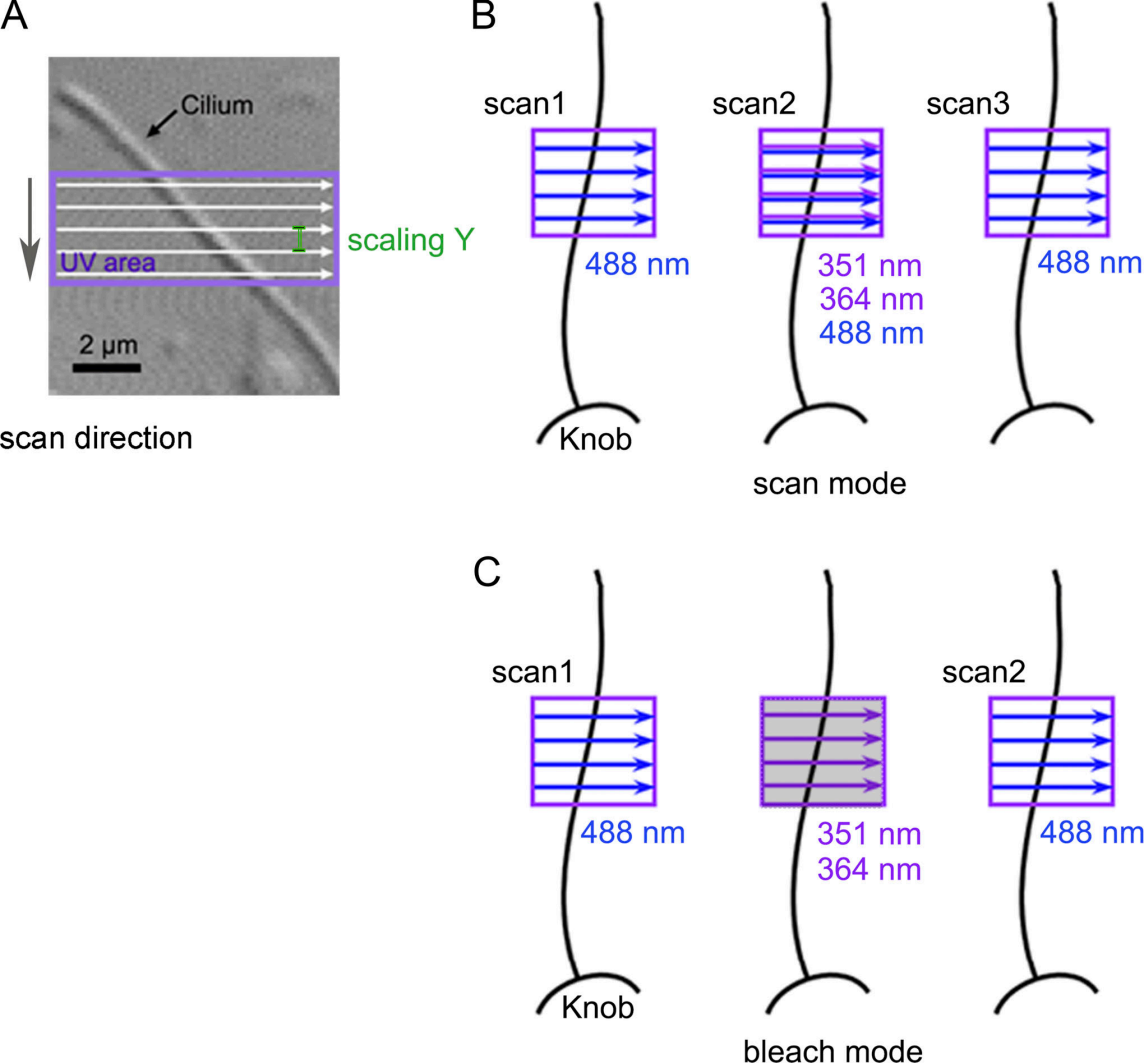
**UV application by scan mode and bleach mode. (A)** Photomicrograph of a single cilium with Nomarski optics and the ROI of the UV irradiation area. The laser beam moves unidirectionally (white arrows) from left to right in the selected area (purple square) within the ROI. The direction of the line-by-line scan was from top to bottom in the scan area as shown by the gray arrow. The distance between the y-axis steps was determined by laser application scaling Y (green). **(B)** Scan mode. We inserted a UV component together with the image scan. **(C)** Bleach mode. The image scan and UV stimulation were independent. In this mode, the line sum parameter is ignored. Images cannot be obtained during UV stimulation (middle in C, shadow).

In the present study, we applied the UV stimulus to a local region of the cilium, and the same (or broader) area was raster-scanned for Ca^2+^ imaging (Fig. 1 A). For all experiments, the transmission parameters for lasers were 100%. We arbitrarily set the line sum parameter in the LSM system to obtain fine images. When this parameter was set to more than one, the line scan was repeated repetitively for that number of times. Specified sum numbers are shown in the figure legends.

Depending on the purpose, we used two different methods for these mixed stimuli and image scans. The first was to include the UV component during the raster scan for Fluo-4 excitation (Fig. 1 B), called the scan mode. This method was appropriate for obtaining Ca^2+^ images simultaneously with UV stimulation. However, one limitation of this technique was that the movement of the beam was constant. The efficacy of the laser beam was determined by the time integral of the applied laser for both photolysis and imaging. In the present experimental system, the intensity required for photolysis was generally higher than that required for imaging. Therefore, a fast scan for imaging could not be achieved using this experimental mode. As an alternate method, we used bleach mode (Fig. 1 C), wherein the UV stimulus could be applied to any region, at any timing, and for any duration as the parameters could be independently set for stimulation and imaging. Therefore, the stimulus intensity was specified by the necessity for UV photolysis, even when a fast image scan was required. One disadvantage was that this mode could not obtain images during UV stimulation (a shadow image in the middle, Fig. 1 C). During breaching in this mode, the sum parameter is ignored.

### Ca^2+^ imaging

An LSM system equipped with an α Plan Fluar (differential interference contrast) 100×/1.45 numerical aperture (oil immersion) objective lens and an argon laser beam (λ = 488 nm; Coherent) was used for the visualization of [Ca^2+^]_i_ using Fluo-4 (F14200; Invitrogen; Thermo Fisher Scientific). A beam splitter (HFT 488) and a long-pass filter (LP 505) were selected. Fluo-4 was initially dissolved in dimethyl sulfoxide and stored at −20°C in complete darkness. The stock was diluted with a Cs^+^-containing pipette solution together with caged cAMP before each experiment. The final concentration of Fluo-4 in the recording pipette was 50 µM. The addition of Fluo-4 did not cause remarkable changes in the membrane current response induced by the cytoplasmic photolysis of caged cAMP, either in time course or degree of adaptation and recovery from adaptation, which is influenced by cytoplasmic Ca^2+^ dynamics. This observation was made based on data comparisons from this study with those from Kurahashi and Menini (1997) and Takeuchi and Kurahashi (2008). Therefore, cytoplasmic Ca^2+^ dynamics are thought to be nearly identical to standard conditions, which have been used for studying ORCs. We did not lower the concentration of Fluo-4 because some cilia were not remarkably stained, even with 50 µM (see Results). Moreover, the concentrations of caged cAMP and Fluo-4 in the cilia may not have been similar to those in the pipette because the recording pipette was placed on the terminal swelling (olfactory knob) or dendrite. It was possible that the concentration in the cilia was lower than the concentration in the pipette.

Although cilia were attached to the bottom of the culture dish, they were randomly winding. If the direction of the laser scanning lines was arranged in a near-parallel position against the longitudinal ciliary axis, then, presumably, the imaging data could be obtained from different areas and UV stimuli could be applied to several regions. Consequently, the data analysis and interpretation would become extremely complicated. Therefore, in this study, scanning lines for both imaging and photolysis were arranged nearly perpendicularly to the cilium to illuminate it only once during a line scan (Fig. 1). LSM 510 software and LSM Image Browser ZEN 2009 (Carl Zeiss Microimaging GmbH) were used for image data acquisition and image processing.

### Fluorescence intensity

**Figure 2.**
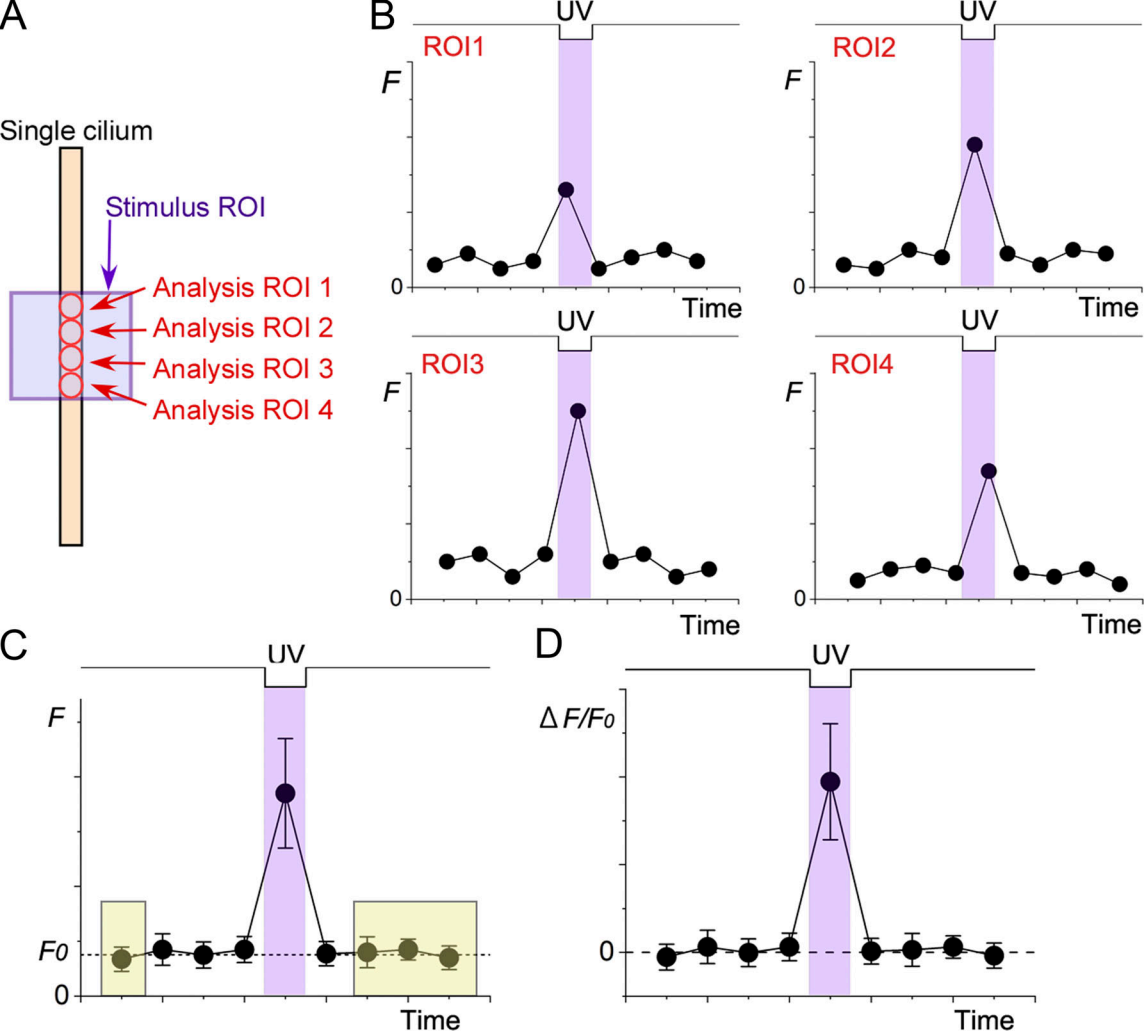
**Measurement and averaging of fluorescence intensity. (A)** Selection of ROI for scan in a single cilium. The UV stimulation area (stimulus ROI) is shown with a purple square. For UV stimulation, scan mode or bleach mode was selected. Analysis ROIs (red circles) were selected along the single cilium after the image was obtained with an excitation laser (488 nm). **(B)** Fluorescence intensities in each analysis ROI were obtained as *F* and plotted against time. Note that the timings for measuring *F* differ depending on the location. Data points are randomly fluctuating values as a model, not the real data. **(C)** The average of the *F*. Error bars are the SD. The timing was set to the middle of the ROIs. *F*_*0*_ was obtained as an average from the data points of the basal level (e.g., yellow shadows). **(D)** *ΔF/F*_*0*_ calculated from C. *ΔF* is *F − F*_*0*_.

**Figure 3.**
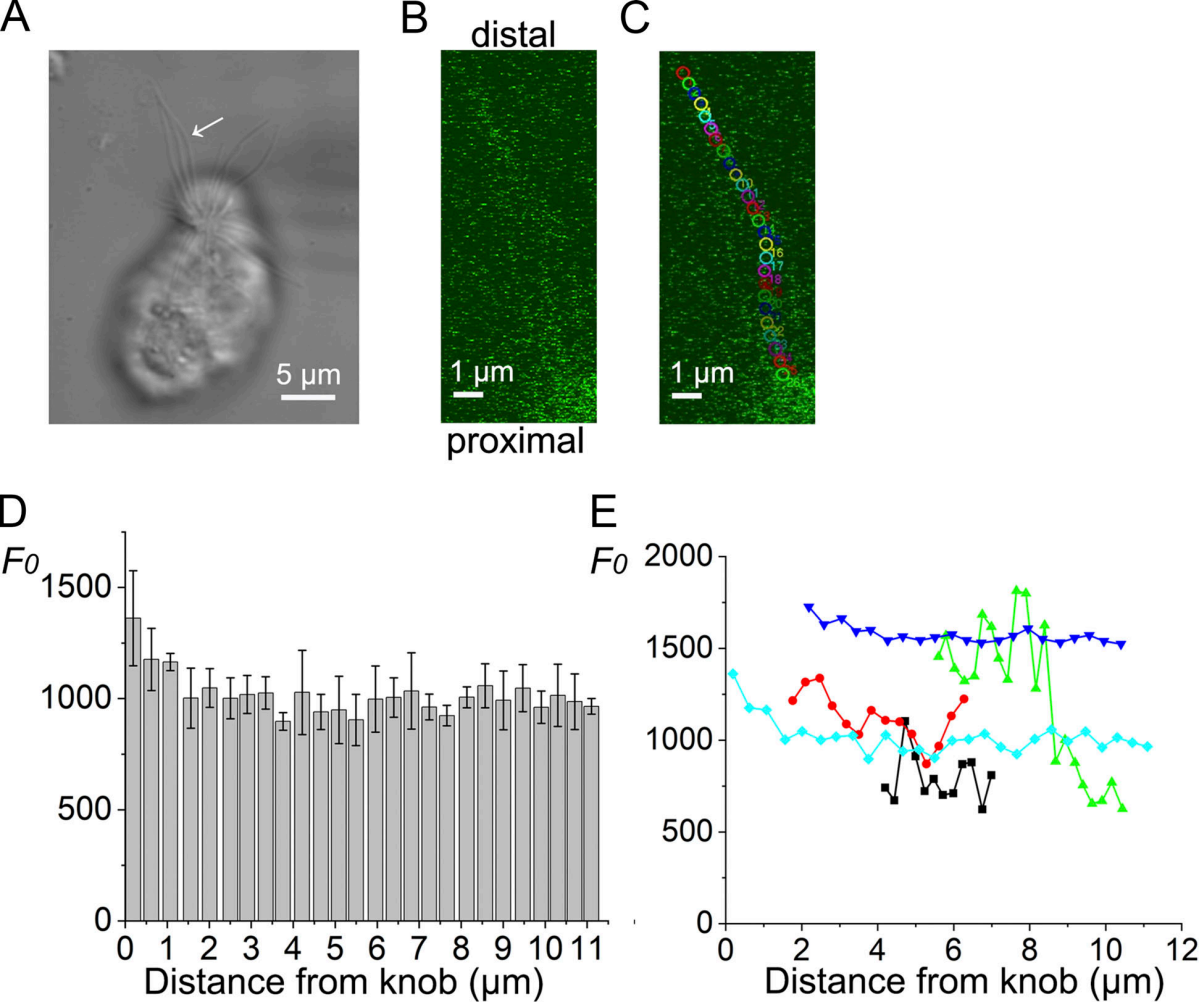
**Basal fluorescence. (A)** Photomicrograph of a single ORC. The blurred recording pipette can be recognized on the right. The arrow indicates the target cilium. **(B)** Fluorescence image of a single cilium indicated in A. Line sum, 2. **(C)** Positions of analysis ROIs (colored circles, 0.41-µm diameter) used for fluorescence measurements. Scaling Y, 0.045 µm. Scan speed, 1.09 s/scan. **(D)** Relation between the basal fluorescence intensity (*F*_*0*_, 12-bit resolution) and the distance from the knob. Data bars are the mean of six plots (technical replicates) during the time sequence and error bars the SD. **(E)** Superimposition of data from five different cells (biological replicates). Background and autofluorescence were not subtracted.

In this study, unless otherwise indicated, we present data using *ΔF/F*_*0*_ (Fig. 2) as an index for the change in [Ca^2+^]_i_. First, we set the analysis ROIs (Fig. 2 A) and obtained fluorescence intensity (*F*, 12 bits) from each area (Fig. 2 B). The background and autofluorescence obtained from the image outside the cilia were subtracted. Second, we calculated the average for *F* and obtained basal fluorescence (*F*_*0*_) from the data points unrelated to the stimulation (Fig. 2 C). The timings for fluorescence measurements were different depending on the position of the analysis ROIs because the excitation of Fluo-4 was done by the raster scan (see Fig. 2 B, peak times are slightly different between ROIs). After averaging the data points, the time was set to the middle of the data points. One may think that averaging *F*_*0*_ within the stimulus ROI causes an error because there is a slight gradient of *F*_*0*_ along the cilium (see Fig. 3 and Results). However, the area of the stimulus ROIs was small, ruling out the effects of *F*_*0*_ gradients. Rather, averaging effectively reduced the noise contained in the fluorescence signal. Finally, we obtained *ΔF/F*_*0*_ from the *F* plots (Fig. 2 D). Fluorescence response was considered significant when the intensity of the stimulus was three times the SD above the basal value. When the *F*_*0*_ gradient was caused by the basal [Ca^2+^]_i_, it was important to analyze the absolute fluorescent intensity *ΔF.* In all the experiments, we also plotted *ΔF* for analyses and saw that the conclusions were the same.

### Timing for UV stimulation

Imaging scans were conducted with an LSM-controlling personal computer (ESPRIMO, P900; Fujitsu) that was independent of the workstation computer (HP xw8600 Workstation; Hewlett-Packard) used for electrophysiology. In previous studies that applied UV stimuli on the ORCs, we included a 488-nm laser together with a UV component (see e.g., Takeuchi and Kurahashi, 2008) to monitor the timing of the UV stimuli. However, in this study, we could not use it that way because we continuously applied a 488-nm laser for Fluo-4 excitation. Therefore, the UV stimulus timings indicated in the figures were estimated from the time of processing of imaging scans that actually included information about the scan process and UV applications. As both the current recording and Ca^2+^ imaging were started independently and manually, a slight error in the timing of the UV application could have been incurred, especially in the current recording.

### Statistical analysis

The current responses and fluorescence intensities were analyzed by a computer and plotted using Microcal Origin 8.6 or 2020 (OriginLab). Data were presented as mean ± SD for the number of experiments indicated. We noted high fluctuation in *F*_*0*_ (see Results), presumably because signals were observed within very small ROIs. Therefore, we averaged data from several areas (Fig. 2).

### Online supplemental material

**Figure S1.**
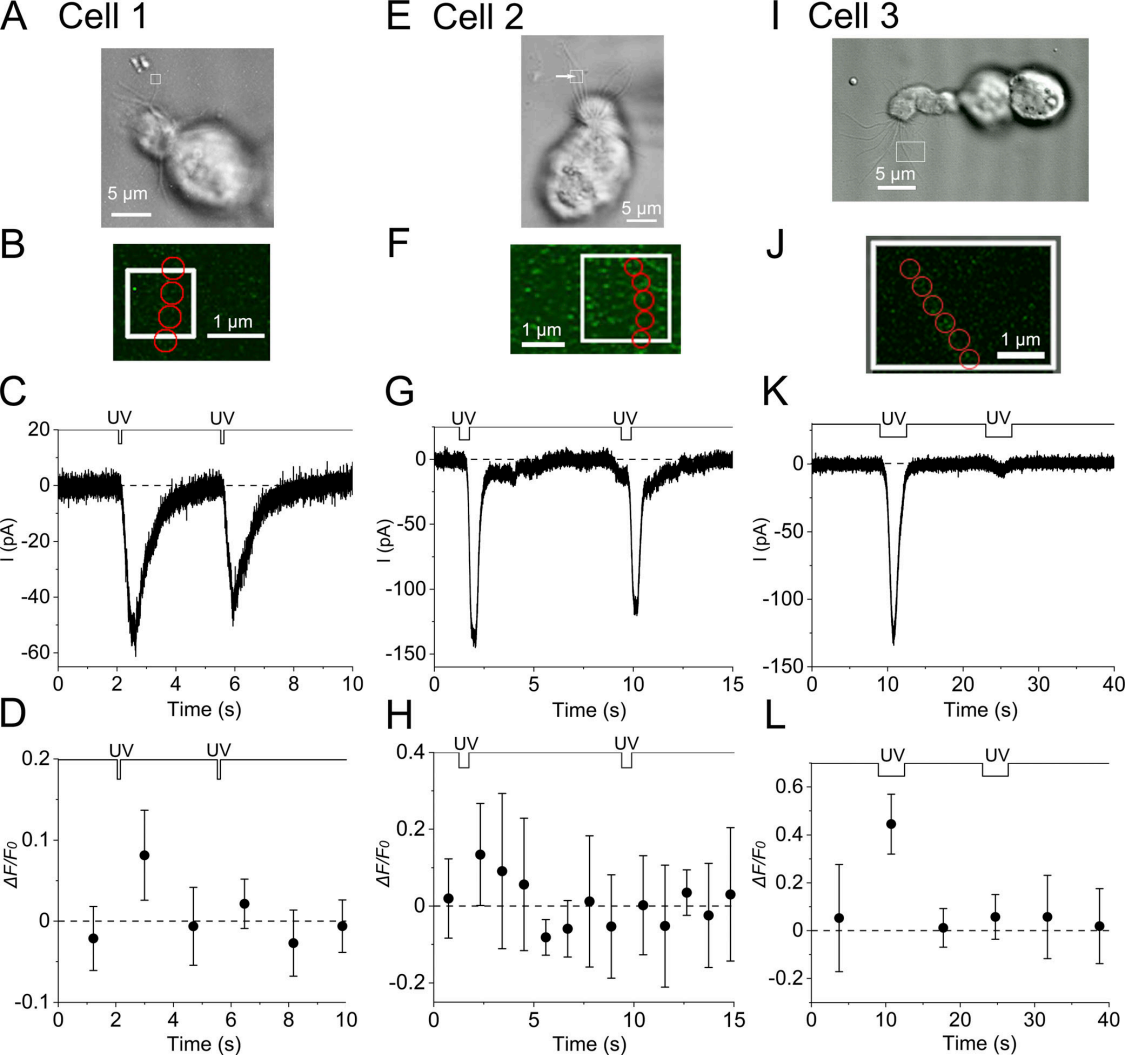
**Time courses of current responses and fluorescence intensities during the adaptation in three cilia from different ORCs.** Data presented in A–D are from the same cilium. **(A)** Photomicrograph of a single ORC (cell 1). The white square shows the stimulus ROI. **(B)** Positions of analysis ROIs (four red circles, 0.41 µm in diameter). Bleach mode. Scan speed, 0.11 s/scan. Interval, 1.70 s/scan. Scaling Y, 0.045 µm. Line sum, 4. **(C)** The current response to UV irradiation. Stimulus duration, 0.11 s. **(D)** Fluorescence intensity changes in the cilium. Plots are means of fluorescence intensities that were obtained from four analysis ROIs. Error bars show the SD from those data. *F*_*0*_ was obtained from data points containing plots outside this time frame (also for H and L). Data in E–H are from the same cilium. **(E)** Photomicrograph of a single ORC (cell 2). Stimulus ROI was selected as indicated with a white square. The same cell as in Fig. 3 A, but the fluorescence was measured in a different cilium (white arrow). Stimulus ROI may have included multiple cilia and the current response may consist of responses from those cilia. However, our conclusions are not affected by such possibilities. **(F)** Positions of analysis ROIs (five red circles, 0.41 µm in diameter). Bleach mode. Scan speed, 0.50 s/scan. Interval, 1.09 s/scan. Scaling Y, 0.045 µm. Line sum, 2. **(G)** The current response to UV irradiation. Stimulus duration, 0.50 s. **(H)** Fluorescence intensity changes in the cilium. Plots are means of fluorescence intensities that were obtained from five analyses ROIs. Error bars show the SD from those data. Data in I–L are from the same cilium. **(I)** Photomicrograph of a single ORC (cell 3). Stimulus ROI was selected as indicated with a white square. It may have included two cilia and the current response may consist of responses from those cilia. However, our conclusions are not affected by such possibilities. **(J)** Positions of analysis ROIs (six red circles, 0.37 µm in diameter). Scan mode. Scan speed, 3.52 s/scan. Interval, 7.00 s/scan. Scaling Y, 0.045 µm. Line sum, 1. **(K)** The current response to UV irradiation. Stimulus duration, 3.52 s. In the data, the current response to the second stimulus decreased to indicate adaptation, but the rate of decrease is very large. The decrease may have several reasons, including adaptation. **(L)** Fluorescence intensity changes in the cilium. Plots are means of fluorescence intensities that were obtained from six analyses ROIs. Error bars show the SD from those data.

Fig. S1 shows the three other different responses by double-pulse UV stimulation.

## Results

### Responses induced by local UV stimulation of a single cilium

**Figure 4.**
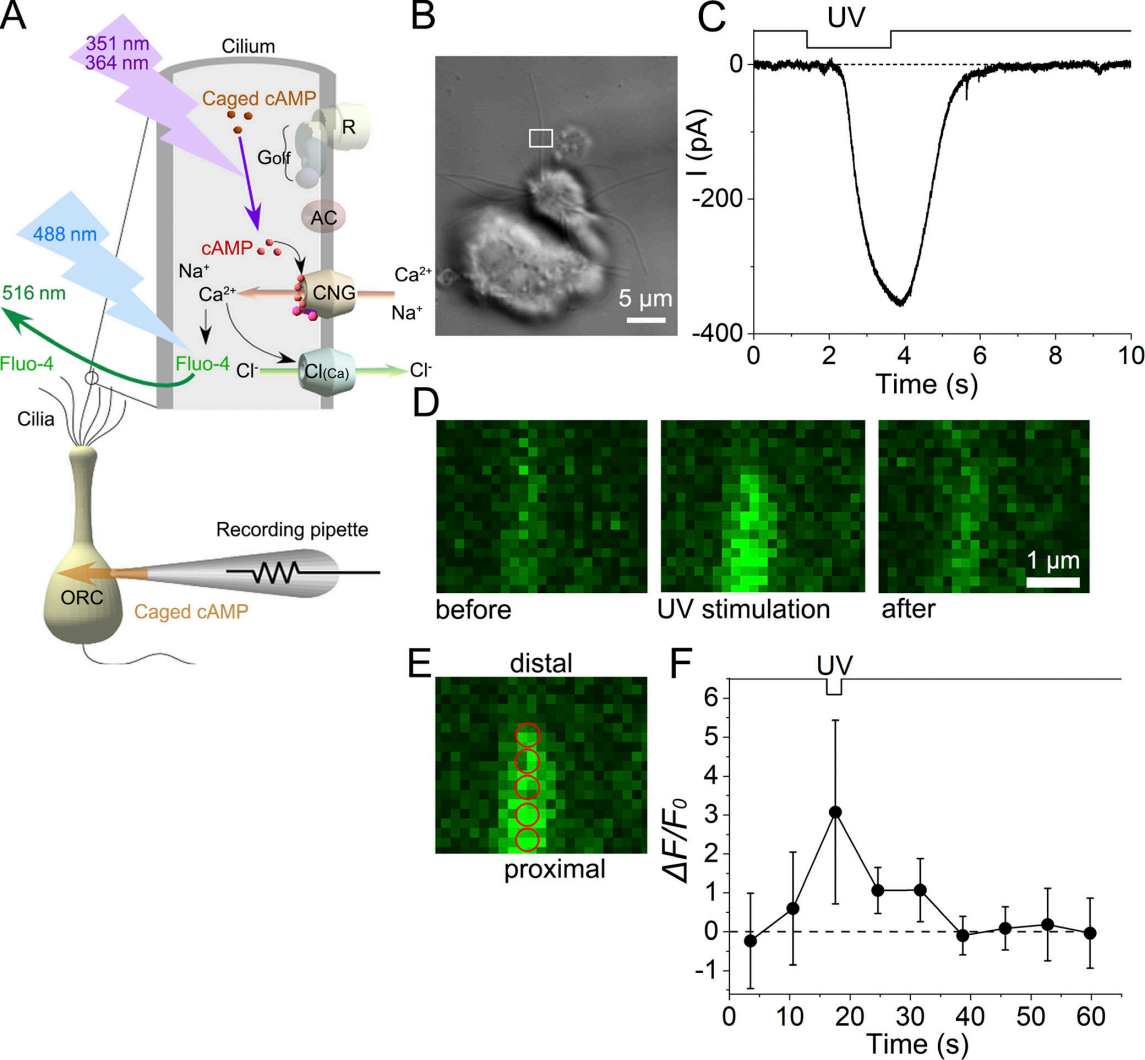
**Current response and fluorescence intensity under scan mode. (A)** Scheme of olfactory transduction and experimental procedures. **(B)** Photomicrograph of a single ORC. The white square shows the ROI area where UV stimulation was applied (stimulus ROI). Fluo-4 fluorescence was obtained from a much larger area in the experiment, but only data from the UV stimulus region are illustrated. **(C)** The current response to UV stimulation. The downward deflection of the upper trace indicates the period of UV stimulation. Duration: 2.22 s. This value represents the time needed for the raster scan of the ROI area. The actual UV application to the cilium comprises repetitive steps (Takeuchi and Kurahashi, 2008). **(D)** Fluorescence images. Left, before UV application; middle, during UV application; right, after UV application. Scaling Y, 0.09 µm. Scan speed, 2.22 s/scan. The intervals between image scan initiations were 7.04 s. Line sum, 4. Note that the increase in fluorescence is not obvious at the beginning of irradiation but becomes more noticeable in the lower region of the middle image. Three images correspond to the second, third, and fourth data points in F. **(E)** Positions of five analysis ROIs (red circles, 0.23 µm in diameter) used for fluorescence measurements. **(F)** Change in *ΔF/F*_*0*_. Plots were obtained from the five analysis ROIs indicated in E. Error bars show the SD from those data.

In this study, we monitored Ca^2+^ dynamics using a Ca^2+^-sensitive dye (Fluo-4) in specific, localized areas of single cilia from the newt while also recording membrane currents using a WC recording configuration. Since CNG channels are Ca^2+^-permeable (see e.g., Kurahashi and Shibuya, 1990), it is expected that local [Ca^2+^]_i_ increases as a result of the opening of CNG channels triggered by the UV laser photorelease of cytoplasmic cAMP (Fig. 4 A).

Before initiating UV stimulation, we evaluated *F*_*0*_ in the cilia using an excitation laser equipped with the LSM system. In 6 of the 19 cilia, we could not detect remarkable staining even with 50 μM Fluo-4 in the patch pipette. In Fig. 3, A–C, an example of cilium that showed remarkable staining by Fluo-4 is presented. When the fluorescence intensity was plotted against the distance from the proximal part of the cilium (olfactory knob), there was a tendency for the fluorescent intensity to be high at the base. It gradually reduced along the longitudinal axis of the cilium (Fig. 3 D). We measured *F*_*0*_ in five cilia that were randomly selected from the stained cells (Fig. 3 E). The degree of fluorescence varied depending on the sample. *F*_*0*_ decreased depending on the distance from the proximal cilia (Fig. 3, D and E). The slope of the fluorescence decrease ranged from −12.77 to −204.62/μm, with a mean and SD of −60.24 ± 81.98/μm (*n* = 5). Although several possibilities exist for such variations in *F*_*0*_ and distance-dependent staining, notably, olfactory cilia are long and conical, with the proximal part being thicker than the apical part (Falk et al., 2015). In addition, it is interesting to see that the samples shown in black and green showed abrupt changes in the *F*_*0*_. This may be related to the presence of microdomains in the olfactory cilia (Castillo et al., 2010) and their variations in Ca^2+^ buffering and/or extrusion capacities. In the present study, however, we did not examine this possibility further.

When the local cilium that contained caged cAMP was stimulated with a raster scan of the UV laser in scan mode, an inward current gradually developed (Fig. 4, A–C). After cessation of the stimulus, the current gradually decreased. Simultaneously, the intensity of Fluo-4 fluorescence increased within the UV-stimulated region of the cilium. For the experiment shown in Fig. 4, the scanning line was moved horizontally, left to right, and the line was repositioned, stepwise, from top to bottom. At the beginning of UV stimulation, no remarkable increase in fluorescence was observed. However, as the scanning time progressed, the fluorescence intensity gradually increased (Fig. 4 D, middle). When the laser raster-scanned the cilium along the longitudinal axis, the cytoplasmic cAMP concentration was assumed to increase locally point by point. If the diffusional processes of the underlying molecules were faster than the scanning speed, the cAMP-induced response would not be observed at a subthreshold level because of the spatially linear summation of the subthreshold responses. This could be caused by a remarkably high nonlinearity (Hill coefficient of > 5) of the olfactory response in single ORCs (Takeuchi and Kurahashi, 2002). However, it was shown in the results that the response increased additively as the laser line repositioned along the cilium (Fig. 4 D, middle). This outcome may suggest that the molecules remained in the vicinity of the stimulation (Takeuchi and Kurahashi, 2018).

**Figure 5.**
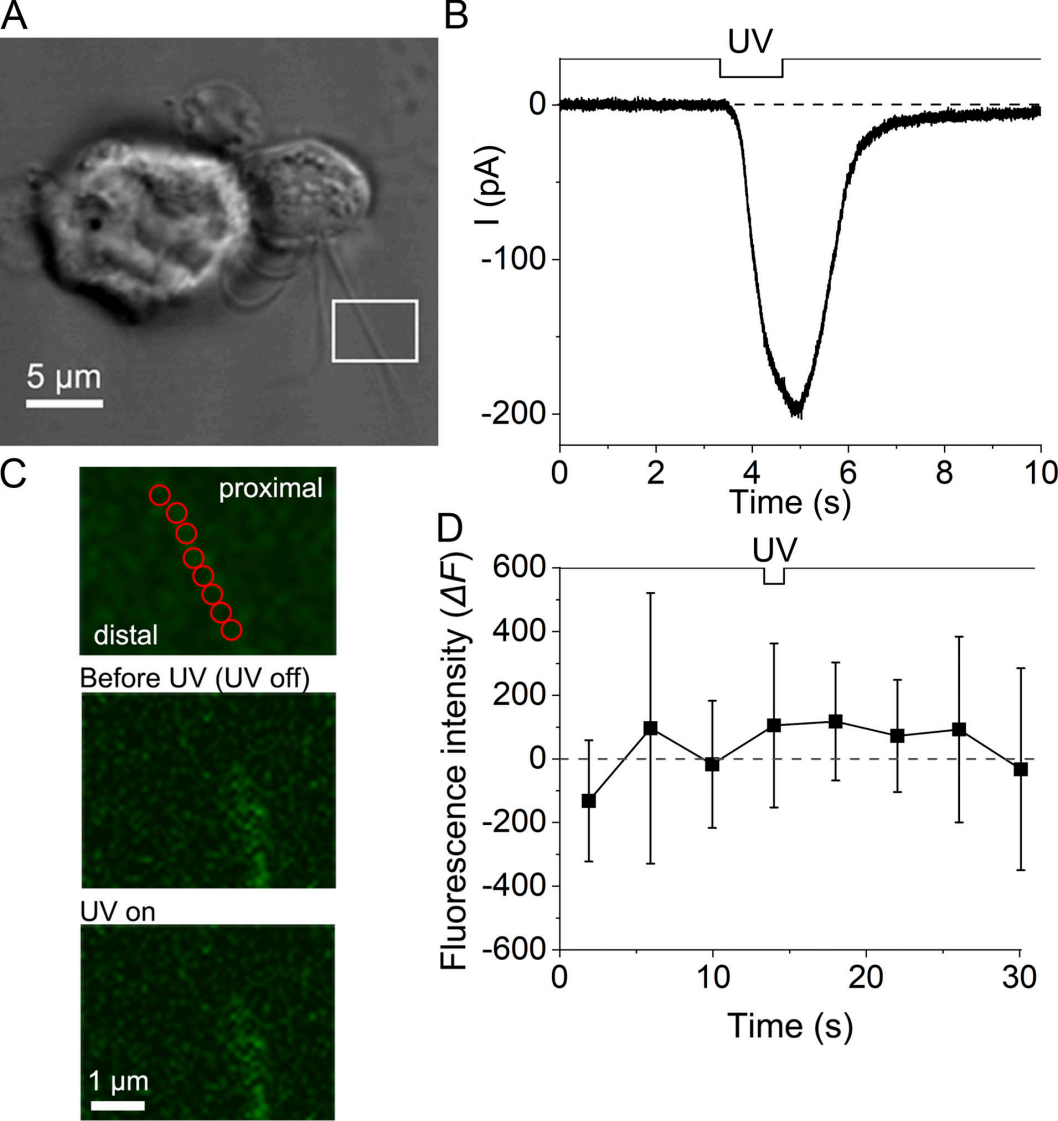
**Cilium showing no remarkable fluorescent response. (A)** Photomicrograph of a single ORC. The white square shows the ROI area where the UV stimulus was applied, and the fluorescence signal was measured. **(B)** The current response to a UV stimulus under scan mode; duration, 1.30 s. **(C)** Top: Analysis ROIs, red circles. Middle: Before UV (third plot in D). Bottom: During UV (fourth plot in D). Scaling Y, 0.18 µm. Scan speed, 1.30 s/scan. The intervals between image scan initiations were 4.03 s. Line sum, 2. **(D)** Change in fluorescence intensity (12-bit data); *n* = 8. Plots are means of fluorescence intensities obtained from analysis ROIs in the UV irradiation area (*n* = 8). The current response shown in B was obtained at the timing of UV in this panel. Error bars show the SD from those data.

**Figure 6.**
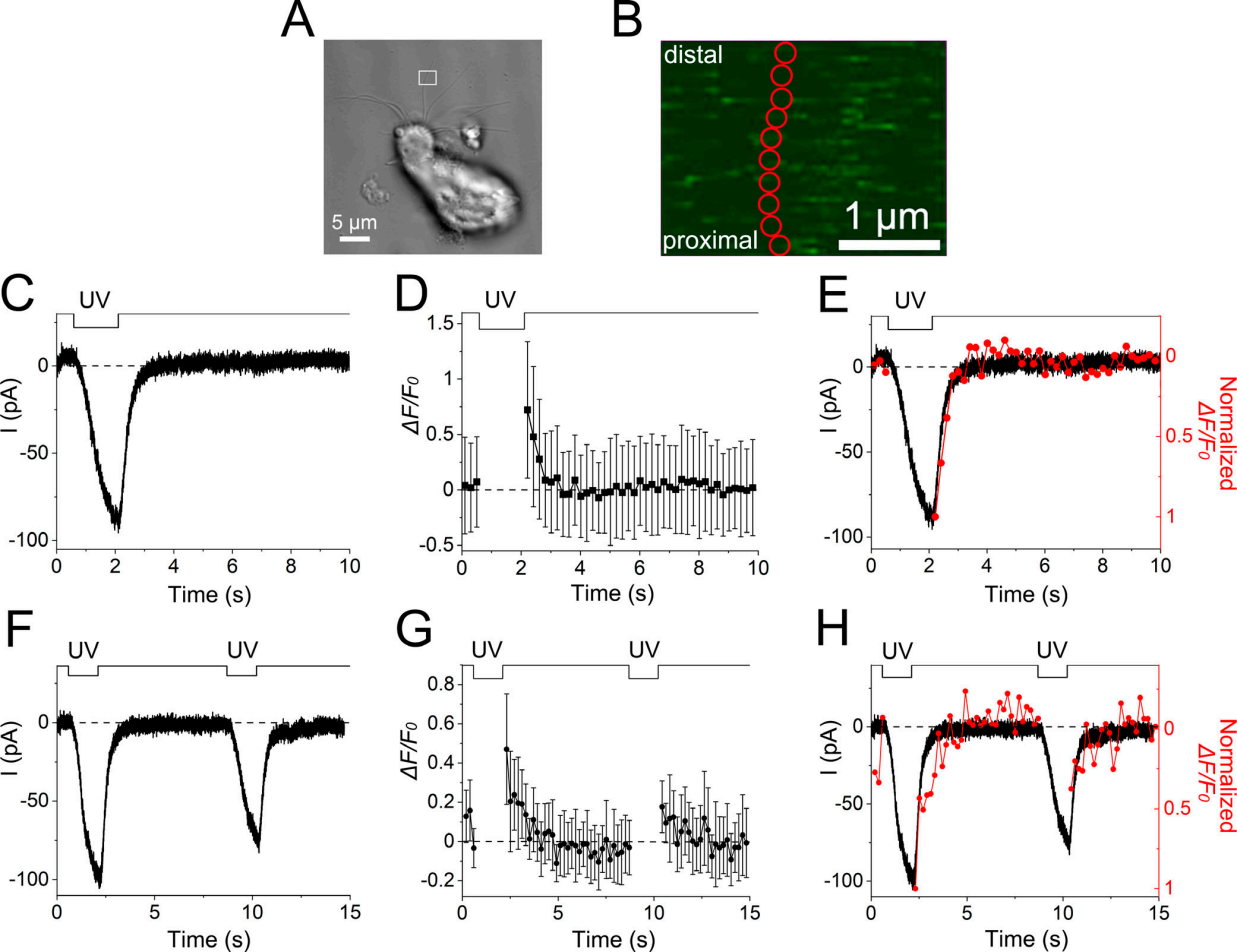
**Direct comparison of time courses for membrane current and Ca**^**2+**^ **signal in the same location of the cilium. (A)** Photomicrograph of a single ORC. The ROI selected for both fluorescence measurements and UV irradiation is indicated by a white square. In this experiment, the ROIs used for imaging and UV stimulation were the same. **(B)** Analysis ROIs on the single cilium (red circles, 0.23 µm in diameter). The total area that appeared in B corresponds to the area marked by a white square in A. Ten small analysis ROIs were used for measuring fluorescence and data obtained from them were averaged. **(C)** The current response to a UV stimulus (1.52 s) using bleach mode (white square in A as a stimulus ROI). Scaling Y, 0.045 µm. **(D)** Fluorescence intensity. Plots are means of fluorescence intensities obtained from analysis ROIs in the UV irradiation area (*n* = 10), as shown in B. Error bars show the SD from those data. Image data and the fluorescence signal during UV irradiation could not be obtained in bleach mode. Scan speed, 0.20 s/scan. Line sum, 2. **(E)** Superimposition of the current response and fluorescence intensity. The data were adjusted by both the peak value and basal level. **(F)** The current response to double-pulse stimulation. **(G)** Fluorescence intensity changes during the double-pulse stimulus. The image scan speed was 0.20 s/scan. **(H)** Superimposition of the current response and fluorescence intensity for the double-pulse stimulus.

When the average fluorescence obtained from five analysis ROIs (Fig. 4 E) was plotted against time, it became clear that the confined UV stimulation induced an increase in fluorescence only after stimulus onset (Fig. 4 F). After its sudden increase, the fluorescence quickly returned to the basal level at the next scan (Fig. 4 D*,* right, and 4 F: see also Fig. 6). Furthermore, the response to UV stimulation could be repeated once the current and fluorescence returned to the background level. The possibility of depletion of exogenous substances in the cilia during the experiments was excluded from this result. Remarkable increases in fluorescence were observed in 9 of the 13 stained cilia preparations upon UV stimulation. The other four cells did not reach the defined criteria for fluorescence response, while they showed remarkable responses in the membrane current (Fig. 5). The following reasons can be attributed to the lack of fluorescence: (1) The introduction of Fluo-4 could be insufficient. Fluo-4 and caged compounds were dissolved in the pipette solution together and introduced into the cilia by free diffusion. Their molecular weights are 478.4 for caged cAMP and 927.1 for Fluo-4, respectively. Thus, it was possible that caged cAMP was introduced to the cilia more efficiently than Fluo-4. (2) The fluorescence change could be smaller than the detection threshold. The signal could be buried in the noise and was not detectable. (3) It was also possible that the depth of the focal plane was slightly different from that of the cilia. Only a 0.1-μm difference causes a significant reduction in the collection of fluorescence because the diameter of the cilia is in that range.

### Comparison of time courses of two responses

The time courses of the current responses and [Ca^2+^]_i_ changes were directly compared within the same cilium with high time resolution (Fig. 6). For these experiments, we used the bleach mode of the LSM system, which can implement independent scan conditions for UV stimulation and imaging (Fig. 1).

Before stimulation, we set the ROI for UV stimulation and imaging (stimulus ROI, white square for bleach stimulation in Fig. 6 A). The analysis ROIs that were used for measuring fluorescence are illustrated with red circles in Fig. 6 B. First, we specified the experimental conditions that induced the inward current response to localized UV stimulation of the cilium by avoiding the saturation of channel activities (Fig. 6 C). Next, we recorded the changes in fluorescence intensities during the current response to the same stimulus conditions (Fig. 6 D). Although we could not monitor the change in fluorescence during the stimulus period under bleach mode, we assumed that the intensity increased. After cessation of the stimulus, we indeed observed an enhancement of fluorescence intensity that gradually returned to the basal level (Fig. 6 D). Falling phases of both responses showed good agreement in the data of Fig. 6 E, but this may have been only a coincidence because both systems contain numerous nonlinear and/or time-dependent processes (see Fig. 6 H, in which time courses are slightly different*,* and see also Discussion). Rather, the experiments notably demonstrated that the Ca^2+^ signal returned to the basal level immediately after the cessation of the UV stimulus.

### Disappearance of Ca^2+^ signals during the adapted state

The return of the Ca^2+^ signal to basal levels with a similar time course as the current response was surprising because Ca^2+^-dependent adaptation has been widely reported to potentially last for a long period even after the current returns to basal levels (Kurahashi and Menini, 1997; Takeuchi and Kurahashi, 2018). We applied double-pulse stimuli and monitored the membrane current (Fig. 6 F) and Ca^2+^ signal to confirm adaptation under this experimental condition (Fig. 6 G). As shown in Fig. 6 F, the second current response was actually smaller than the first one despite them having the same UV stimulus. This is a stereotypical feature of adaptation. For both the first and second responses, the fluorescence intensities followed the current traces (Fig. 6 H). These results clearly indicated that [Ca^2+^]_i_ returned to the basal level before adaptation ended (see potential molecular explanations for this finding in the Discussion).

We could apply double-pulse stimuli to eight ciliary preparations (from different ORCs). Among them, six had appropriate stimulus interval time in investigating adaptation. Five out of six showed remarkable adaptation in current responses (one preparation showed “summation,” see Takeuchi and Kurahashi, 2018). Four of them showed significant fluorescence signals, and all four expressed the recovery in fluorescence intensities to the background before applying the second stimulation (Fig. S1).

### Ca^2+^ dynamics in olfactory cilia at 0 mM EGTA

Under the WC recording configuration, the cytoplasmic environment is influenced by direct interaction with an intrapipette solution through the hole under the tip of the recording pipette. Therefore, in the WC condition of ORCs, 5 mM EGTA is usually used to stabilize the recordings. This environment is expected to potentially imitate well the Ca^2+^ buffering capacity of the cell interior under physiological conditions. However, extrinsic EGTA may cause side effects distinct from the endogenous Ca^2+^ buffers, especially when focusing on the kinetics of Ca^2+^, as in this study. In this work, we also recorded cell responses with a 0 mM EGTA pipette, presumably before the intrinsic Ca^2+^ buffers were washed out. Cell conditions without EGTA are generally known to deteriorate compared with recordings with a 5-mM EGTA pipette, as has been reported in a previous study (Takeuchi and Kurahashi, 2018). This outcome could be caused by a reduced buffering capacity and an additional Ca^2+^ that is included in the nominal Ca^2+^-free solution (Kurahashi, 1990). Although the cytoplasmic buffering capacity is obviously reduced in the 0 mM EGTA condition, such a condition could provide substantial information without adding extrinsic Ca^2+^ buffers.

**Figure 7.**
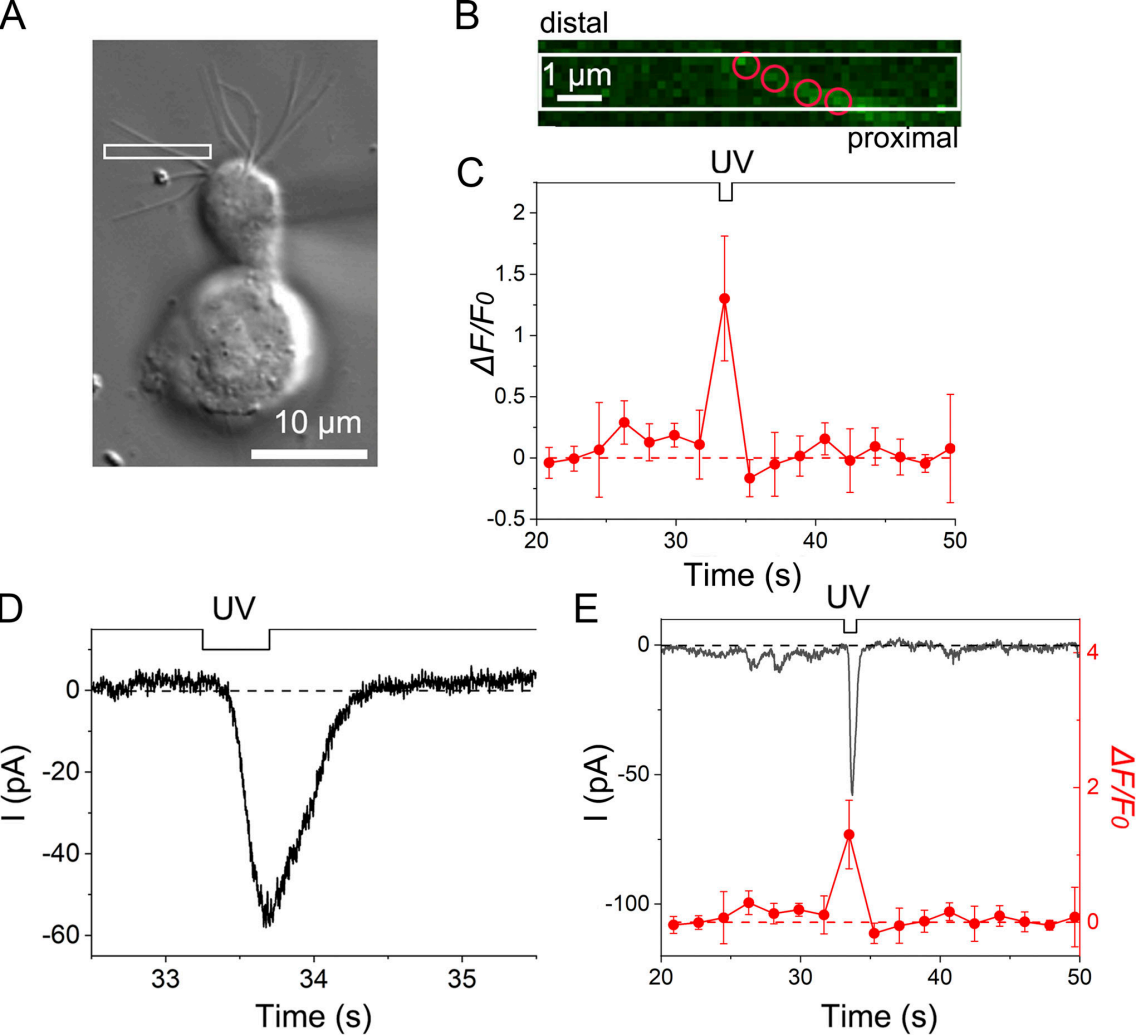
**Ca**^**2+**^ **dynamics in the olfactory cilium in the absence of Ca**^**2+**^ **buffer in the recording pipette. (A)** Photomicrograph of a single ORC. The white square shows the stimulus ROI. EGTA was not added to the recording pipette. **(B)** Positions of analysis ROIs (four red circles). Scan mode. Scan speed, 0.45 s/scan. Interval, 1.80 s/scan. Scaling Y, 0.18 µm. Line sum, 4. **(C)** Fluorescence intensity changes in the cilium. Plots are means of fluorescence intensities that were obtained from four analysis ROIs. Error bars show the SD from those data. **(D)** The current response to UV irradiation. Stimulus duration, 0.45 s. **(E)** Superimposition of current response and fluorescence intensity.

We succeeded in observing stable and remarkable increases in fluorescence in 2 of the 16 cilia preparations in the 0 mM EGTA condition and, subsequently analyzed the Ca^2+^ signal. As in experiments using a 5-mM EGTA pipette, the Ca^2+^ signal increased significantly at the site of UV photolysis (Fig. 7, A–C). At the same time, an inward current response was obtained (Fig. 7 D). Immediately after the cessation of UV stimulation, both the current and Ca^2+^ signals returned to basal levels (Fig. 7 E). Although the imaging rate was slow in the experiment shown in Fig. 7, the fluorescence returned to the basal level in the next measurement after the UV stimulation (i.e., 2 s). In general, adaptation remained during this period.

**Figure 8.**
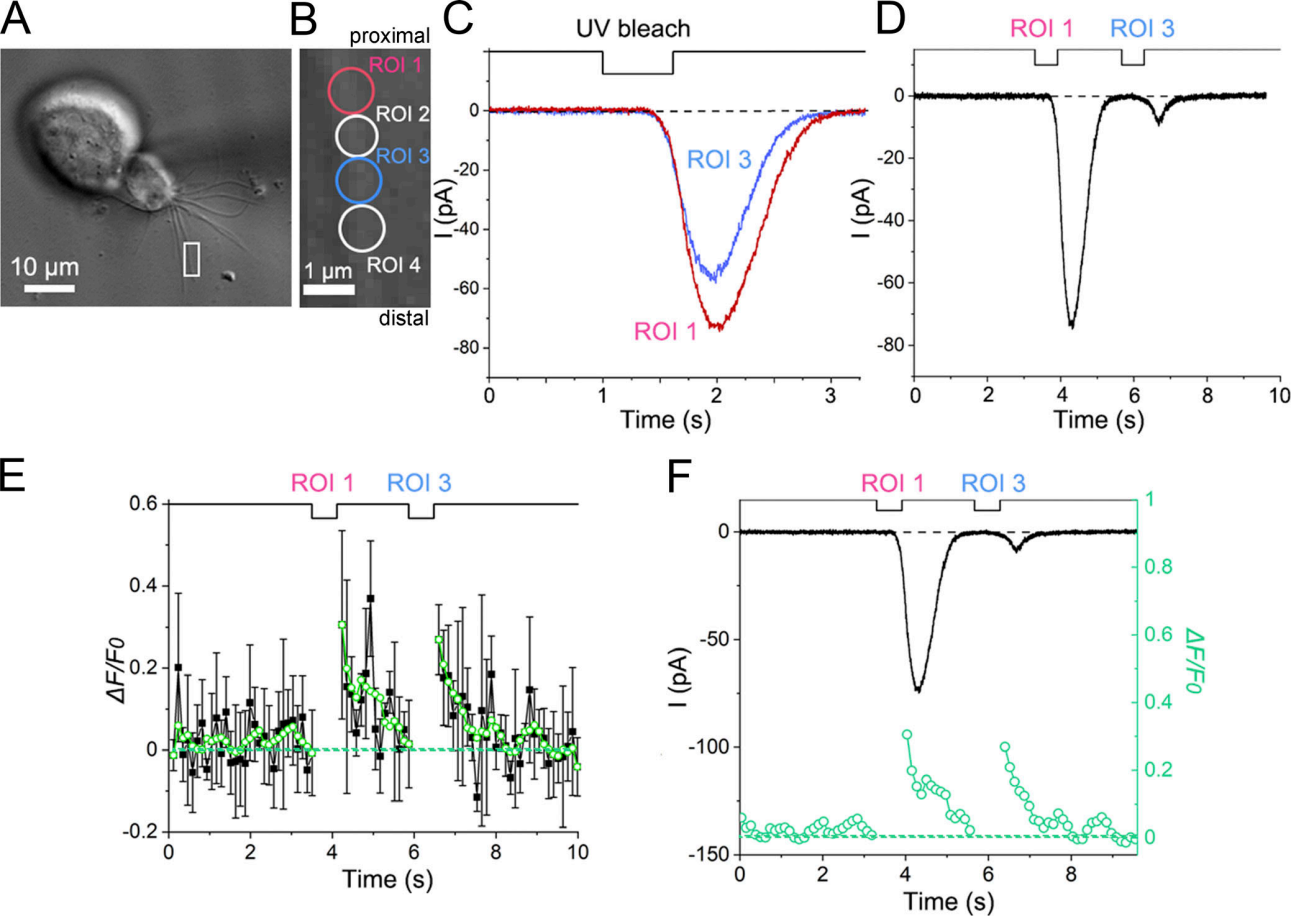
**Adaptation and Ca**^**2+**^ **dynamics as examined in the absence of EGTA in the recording pipette. (A)** Photomicrograph of a single ORC. The ROI scan area is indicated by a white square. Scan speed, 0.12 s/scan with bleach mode. **(B)** Positions of ROIs on the single cilium (circles, 0.9 µm in diameter). Stimulus ROIs were ROIs 1 and 3. Analyses ROIs, ROIs 1–4. **(C)** Single current responses of ROIs 1 and 3. UV bleach duration: 0.62 s for each. Scaling Y, 0.09 µm. Line sum, 1. **(D)** Cross-adaptation. Current responses to double-pulse stimuli. **(E)** Fluorescence intensity changes during the double-pulse stimulus. The first bleach was applied to ROI 1 (red circle in B); the second bleach, to ROI 3 (blue circle in B). Data represented by black squares were obtained as averages from the four analysis ROIs shown in B (red, blue, and two white ROIs, error bars show SD from those data), whereas data represented by green circles were obtained after the adjacent-averaging (moving average) procedure in Origin program. The number of adjacent averaging points of the window was five. **(F)** Comparison of time courses between the current response and fluorescence intensity.

**Figure 9.**
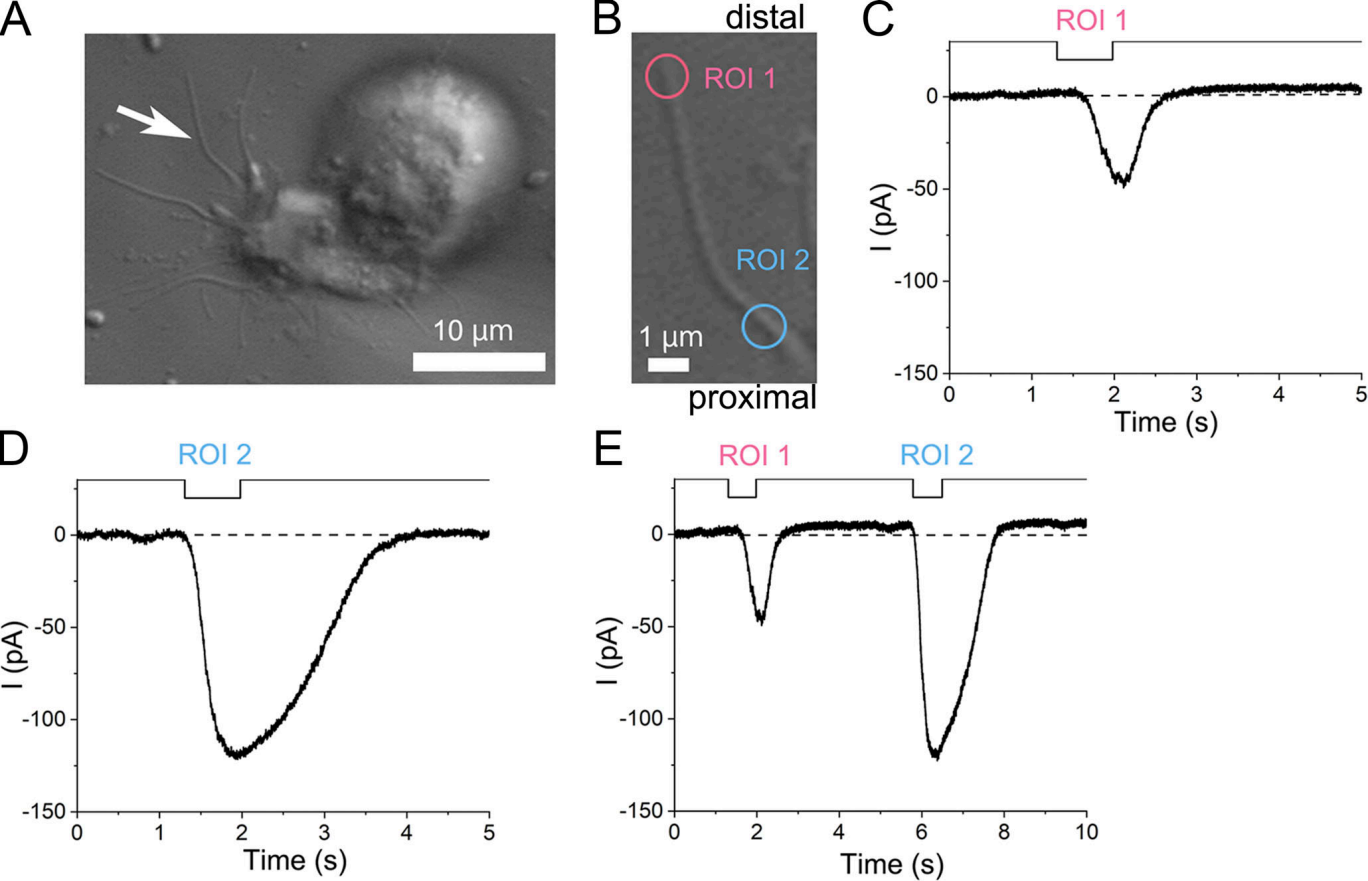
**The lack of adaptation when the distance between the two stimulus sites was far apart (6.4 μm). (A)** Photomicrograph of a single ORC. The white arrow indicates the target cilium. No EGTA was added to the recording pipette. **(B)** Positions of stimulus ROIs on the single cilium (circles, 1.08 µm in diameter). Scaling Y, 0.045 µm. **(C)** The current response to the ROI 1 stimulation only. **(D)** The current response to the ROI 2 stimulation only. **(E)** Current responses induced by double-pulse stimulation. Note that the size of the response induced at ROI 2 is unaffected by a preconditioning pulse applied to ROI 1. A–E are data from the same single cilium.

After obtaining convincing Ca^2+^ responses, we investigated Ca^2+^ dynamics and adaptation in the 0 mM EGTA condition in a stable preparation among two using bleach mode (Fig. 1). Two stimulus ROIs (1 and 3) were set within a close distance on a single cilium (Fig. 8, A and B). When these ROIs were independently stimulated with UV, we observed individual current responses (Fig. 8 C). The response to the UV stimulation to ROI 1 was ∼30% larger than that to the UV stimulation to ROI 3, which was located slightly more distally. Such location dependence of the cAMP response has been reported previously (Takeuchi and Kurahashi, 2008). We next performed a double-pulse protocol between ROIs 1 and 3 with an interval of ∼2 s. The current induced by the stimulus to ROI 3 was remarkably reduced following a preconditioning pulse applied to ROI 1, which is a typical sign of adaptation (Fig. 8 D). It has been shown in previous research that Ca^2+^ can diffuse such a distance even when the two ROIs are slightly separated (Takeuchi and Kurahashi, 2018). This property, typical of adaptation, was not observed when the two stimulus points were far apart (Fig. 9; see also Takeuchi and Kurahashi, 2018). One may think that the direction of the stimulation (e.g., distal to proximal or vice versa) may cause some differences in the expression of adaptation. However, it has been shown that there is no direction dependence on adaptation when examined with double-pulse protocols in olfactory cilia (Takeuchi and Kurahashi, 2018).

Simultaneously, we monitored the Ca^2+^ signal around the stimulus sites (ROIs 1–4). The Ca^2+^ signal also increased after UV stimulation (Fig. 8 E) at both the first and second stimuli. Notably, after the first response, the fluorescence intensity returned to the basal level (Fig. 8 F), whereas adaptation remained persistent. This result is consistent with that observed in the control condition (Fig. 6) and indicated that exogenous Ca^2+^ buffers did not cause significant side effects in concluding Ca^2+^ behavior during adaptation. The size of the increase in fluorescence intensity at the second stimulation may be noted to be almost comparable with that at the first stimulation, despite the significant difference in size between the two current responses. The fraction of the Cl^−^ component may have differed between the first and second current responses, but we did not analyze this matter in further detail.

## Discussion

In this study, local Ca^2+^ signals induced by the UV-laser-stimulated photolysis of caged cAMP and the opening of transducer channels were monitored simultaneously in single olfactory cilia in real time. Our results show that Ca^2+^ signaling is clearly segregated between two opposing functions, even in a very tiny space. Since the same results were obtained in experiments in which no exogenous Ca^2+^ buffer was added, and such segregation is likely performed in native olfactory cilia.

### Disappearance of Ca^2+^ signals during the adapted state

The local region of cilium showed adaptation that was regulated by cytoplasmic Ca^2+^ even after Fluo-4 fluorescence had returned to basal levels (Figs. 6 and 8). Although this occurrence could be ascribed to the adaptation system having a higher Ca^2+^ sensitivity than Fluo-4, this scenario is unlikely. The *K*_d_ value of Fluo-4 is 345 nM (Gee et al., 2000), whereas the *K*_d_ value of calmodulin (CAM), one of the candidates Ca^2+^-binding proteins that could mediate olfactory adaptation, ranges from 4 to 7 μM (Chen and Yau, 1994). Thus, compared with CAM, Fluo-4 likely covers much lower concentrations of Ca^2+^. Incidentally, the *K*_d_ value of Ca^2+^-activated Cl^−^ channels is ∼5 μM (Kleene and Gesteland, 1991; Frings et al., 2000; Billig et al., 2011), which covers almost the same [Ca^2+^]_i_ range as that of CAM.

A more likely and intriguing possibility at play here is that an exclusive interaction between Ca^2+^ and Ca^2+^-binding proteins that mediate adaptation may modulate the adaptation lifetime. A straightforward interpretation is the slow dissociation of the protein. Liu et al. (1994) showed that the CNG channel exhibits an affinity change in response to Ca^2+^–CAM. The recovery of current after exposure of the CNG channel to Ca^2+^–CAM was slower than several seconds (see Fig. 3 in Liu et al., 1994). In cilia, the opening of CNG channels causes a sudden increase in [Ca^2+^]_i_, which would activate Cl^−^ channels, and is detected by Fluo-4. The onset rate of Ca^2+^–CAM effects on CNG channels is also very slow (see Fig. 3 in Liu et al., 1994). A slow onset rate would allow free Ca^2+^ to bind to Fluo-4 and/or Ca^2+^-activated Cl^−^ channels before activating the function of Ca^2+^–CAM. Once Ca^2+^ interacts with Ca^2+^-binding proteins, the free [Ca^2+^]_i_ would be reduced and Ca^2+^–CAM complexes could be maintained for tens of seconds, thereby mediating ciliary adaptation.

Another possibility is a local molecular circuit (Castillo et al., 2010). If the interaction between Ca^2+^ and Ca^2+^-binding proteins is isolated from the bulk cytoplasm, then Fluo-4 would not be able to detect free Ca^2+^ even during adaptation. However, in this case, we could not explain why the Ca^2+^ signals and Ca^2+^-activated Cl^−^ currents were observed when the inward current was flowing.

### Free Ca^2+^ in the ciliary cytoplasm and the time course of Ca^2+^-activated Cl^−^ current

Because Ca^2+^ entry through the odorant-activated cation channel is essential for odor adaptation in the ORC (Kurahashi and Shibuya, 1990), free [Ca^2+^]_i_ has long been believed to be kept high during the adapted state in cilia. However, our results revealed that free [Ca^2+^]_i_ rapidly returned to basal levels even during adaptation. Moreover, it has been shown in electrophysiological analysis that the activity of Ca^2+^-activated Cl^−^ channels is terminated even when adaptation was observed. The time course of the Cl^−^ current is very similar to that of the odorant-triggered cation channel (Kurahashi and Yau, 1993) that is now considered to be identical to the CNG channel (see Schild and Restrepo, 1998; Frings, 2001; Kaupp and Seifert, 2002; Matthews and Reisert, 2003; Trudeau and Zagotta, 2003; Kleene, 2008; Dibattista et al., 2017). Thus, the absolute amplitude of the Cl^−^ current reflects the instant influx of Ca^2+^.

The Cl^−^ channel does not exhibit fast desensitization when examined in an inside-out patch preparation (Reisert et al., 2003; Pifferi et al., 2009), although it does exhibit a slow rundown. The rapid falling phase found in the WC preparation could occur because Ca^2+^-activated Cl^−^ channels could be desensitized when they were situated in the native cilium. In fact, CNG channels do not show desensitization when examined in an excised patch preparation (Kurahashi and Kaneko, 1993). Still, they express adaptation in native cilia (Kurahashi and Shibuya, 1990; Kurahashi and Menini, 1997). However, our finding that free Ca^2+^ itself is quickly abolished supports the notion that Ca^2+^-activated Cl^−^ channels in cilia have little desensitization. Thus, the rapid reduction in the Cl^−^ current that could result from the disappearance of free Ca^2+^ would be a natural thought.

In the data in Fig. 6, the falling phase of the Ca^2+^-activated Cl^−^ current mirrored the reduction time course of the Fluo-4 fluorescence intensity. One may simply consider that the falling phase of the Cl^−^ current represents the reduction of [Ca^2+^]_i_ very precisely. However, this idea could be too speculative at this moment. Cl^−^ current and Fluo-4 signal contain differences in Ca^2+^ sensitivities, namely, (1) nonlinearity of [Ca^2+^]_i_ sensitivities in both systems, (2) differences in *K*_*1/2*_ values, and (3) differences in the time course and spatial distribution. More detailed analyses are needed to understand these possibilities.

### Relationship between washout of cytoplasmic Ca^2+^ buffer and introduction of caged substance plus Fluo-4

Most cells deteriorated progressively after the establishment of the WC recording configuration when pipettes without added Ca^2+^ buffer were used (see also Takeuchi and Kurahashi, 2018). This indicates that the intrinsic Ca^2+^ buffer plays a crucial role in cell survival. Although the success rate was meager, we could record current responses under such conditions. At the same time, in the present experiments, we added caged cAMP and Fluo-4 to the pipette to introduce these substances into the cytoplasm. We assume there is a limited time window in which extrinsic substances become active while intrinsic Ca^2+^ buffers remain.

Usually, after the establishment of the WC configuration, intrinsic Ca^2+^ buffers are washed out and substituted with substances in the recording pipette. One may imagine that the washout of cytosolic factors and the introduction of exogenous substances co-occur. However, we must interpret data regarding differences between washout and the introduction of factors. For instance, diffusion could differ between intrinsic Ca^2+^ buffers and caged substances plus Fluo-4. Cytosolic Ca^2+^ buffers are usually proteins that are larger than the introduced molecules.

Furthermore, diffusional processes may be related to the spatiotemporal distribution of substances in the cilia of the ORC. Ca^2+^ buffers could be strong in the cell body because [Ca^2+^]_i_ is strongly related to cell survival. On the other hand, the patch pipette was placed near the dendritic terminal where the cilia extend. It was possible in a small number of cells examined that caged substances and Fluo-4 diffused rapidly into the ciliary cytoplasm.

### Number of Ca^2+^ molecules and their extrusion

Intracilial Ca^2+^, which enters through the CNG channel, must be excluded from the cytoplasm because the Cl^−^ current is terminated (McClintock and Ache, 1990; Kurahashi and Yau, 1993; Li et al., 2016) and adaptation becomes gradually nonfunctioning (Kurahashi and Menini, 1997). Ca^2+^ extrusion could occur through several candidates in the cilium (see Pifferi et al., 2010), such as the Na^+^/Ca^2+^ exchanger (Reisert and Matthews, 1998; Pyrski et al., 2007; Stephan et al., 2011; Ferguson and Zhao, 2017) and/or ATP-dependent Ca^2+^ pump (Castillo et al., 2007). However, the spatial distribution of Ca^2+^ extrusion systems has not been discussed in detail.

Together with a previous study (Takeuchi and Kurahashi, 2018), our findings revealed that cytoplasmic Ca^2+^ did not diffuse very far from the site of influx. As short-term adaptation is conducted exclusively by Ca^2+^ in the ciliary cytoplasm (Kurahashi, 1990; Kurahashi and Shibuya, 1990), Ca^2+^ would be extruded locally during the recovery phase of adaptation. As described, the recovery time course from adaptation is much slower than the termination of the transduction current that returns to the resting level within a few seconds. In contrast, adaptation lasts for >10 s (Fig. 6; see also Kurahashi and Menini, 1997).

We can roughly estimate [Ca^2+^]_i_ because Ca^2+^-activated Cl^−^ channels exhibit saturation during the odorant-induced response (see Fig. 6 of Li et al., 2016). When examined with a detached cilia preparation, Ca^2+^-activated Cl^−^ channels are saturated at 20–30 μM (Kleene and Gesteland, 1991). Therefore, [Ca^2+^]_i_ can be higher than these values. Assuming that [Ca^2+^]_i_ is 100 μM, the number of Ca^2+^ molecules is 4.7 × 10^2^ for every micrometer of a cilium. Extrusion of these Ca^2+^ within 10 s results in 4.7 × 10 molecules/s, which can be established even by very slow transporters. The CNG channel, which allows Ca^2+^ influx across the plasma membrane upon odorant stimulation, was previously shown to be distributed throughout the cilium (Takeuchi and Kurahashi, 2008). As CAM is associated with the CNG channel (Bradley et al., 2001), adaptation occurs throughout the cilium. Ca^2+^-activated Cl^−^ channels are also distributed evenly, spanning the cilium (Takeuchi et al., 2009). Moreover, second messenger molecules have a limited spread in olfactory cilia (Takeuchi and Kurahashi, 2018). Therefore, the Ca^2+^ extrusion system is also likely widely distributed throughout the cilium.

It has been shown in studies on rod photoreceptor cells that Na^+^/Ca^2+^/K^+^ exchange is electrogenic and that Ca^2+^ movement can be detected as electrical activity (Yau and Nakatani, 1984; Perry and McNaughton, 1993). Because one Ca^2+^ and one K^+^ are pumped out, whereas four Na^+^ molecules are flowing in, the number of charge movements is identical to the number of Ca^2+^ molecules extruded. However, reports on olfactory cilia showing electrical signs accompanying Ca^2+^ extrusion during or after the odorant response are lacking. We hypothesized that much of Ca^2+^ is extruded from the ciliary membrane during recovery from adaptation, presumably immediately after dissociating from the binding protein.

Therefore, one may speculate that charge movement can be detected using an electrophysiological technique. Charge movements accompanying Ca^2+^ extrusion across the ciliary membrane are calculated to address this speculation. As discussed, the number of Ca^2+^ molecules involved in the odorant response could be 4.7 × 10^2^/μm cilium (100 μM); if this number is extruded in 10 s, then the average current will be 7.6 × 10^−6^ pA (7.6 aA)/μm cilium. If the length of the cilium is 10 μm and the total number of cilia is 10, then the current amplitude in an ORC becomes 0.76 fA, which cannot be detected using conventional electrophysiology.

Thus, olfactory signal transduction and its modulations are efficiently performed with a very small number of molecules. An extremely high surface-to-volume ratio unique to nanoscale tubular structures such as cilia is highly likely to be at least partially responsible for such specific purposes (Takeuchi and Kurahashi, 2008; Takeuchi and Kurahashi, 2018). Methodologies and new concepts can be used to investigate further the mechanisms of olfactory signal transduction and those of other systems performed in submicron spaces.
